# Intranodal Lymphangiography during Surgical Repair of Pelvic Lymphorrhea after Radical Cystectomy

**DOI:** 10.1155/2021/7822422

**Published:** 2021-07-05

**Authors:** Yasuyuki Onishi, Yusaku Moribata, Hironori Shimizu, Kosuke Shimizu, Takeshi Sano, Takashi Kobayashi, Yuji Nakamoto

**Affiliations:** ^1^Department of Diagnostic Imaging and Nuclear Medicine, Kyoto University Graduate School of Medicine, Japan; ^2^Department of Urology, Kyoto University Graduate School of Medicine, Japan

## Abstract

Lymphorrhea can develop after various types of surgeries. Surgical closure of the lymphatic leakage point is an effective treatment option. However, it is difficult to identify the leakage point sometimes. Here, we report a case of pelvic lymphorrhea after radical cystectomy for bladder cancer. Identification of the leakage point was difficult during laparoscopic surgical repair of lymphorrhea. Intranodal lymphangiography was performed via the inguinal lymph node by injection of lipiodol, followed by injection of indigo carmine. Laparoscopy revealed extravasation of lipiodol and indigo carmine from the pelvic wall. The leakage point was successfully cauterized using an electric scalpel. Lymphorrhea improved after the surgical repair. This case suggests that intranodal lymphangiography may be useful for detecting the site of lymphatic leakage during the surgical repair of lymphorrhea.

## 1. Introduction

Lymphorrhea can occur after various types of surgeries, including esophagectomy, pneumonectomy, gastrectomy, pancreaticoduodenectomy, kidney transplantation, hysterectomy, prostatectomy, and lymph node resection [1]. Dehydration, nutritional deficiency, and immunological dysfunction may develop due to lymphorrhea [2]. Conservative management, such as dietary therapy, total parenteral nutrition, pharmacotherapy, and abdominal paracentesis, is an effective initial treatment [3]. If conservative management fails, surgical closure of the site of lymphatic leakage is the ideal treatment [3, 4]. However, the leakage point is occasionally not identified during surgery [3]. Intraoperative lymphangiography is an effective method for identifying the leakage point. Herein, we describe a case of postoperative lymphorrhea that was treated with surgical repair. Intranodal lymphangiography was performed during the repair to identify the site of leakage.

## 2. Case Presentation

**Figure 1:**
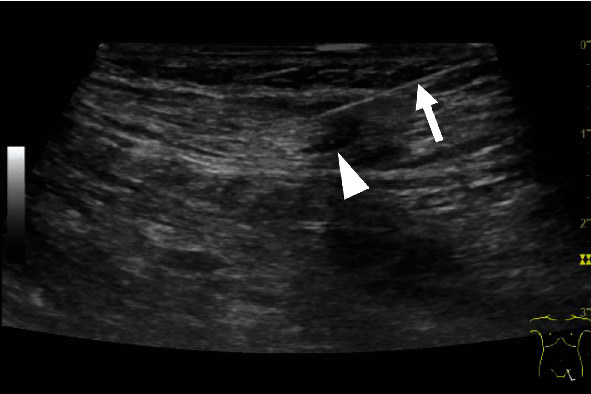
Ultrasonography performed during intranodal lymphangiography demonstrating a 23-gauge needle (arrow) being advanced into an inguinal lymph node (arrowhead).

**Figure 2:**
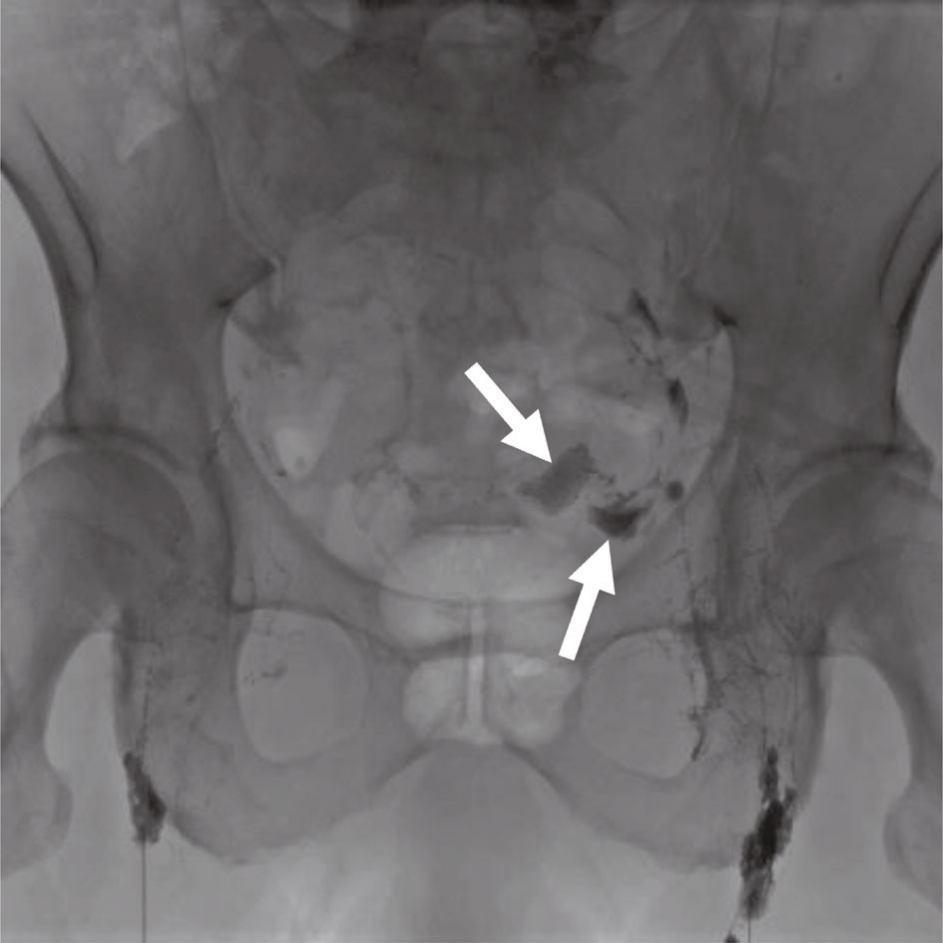
Pelvic X-ray performed during intranodal lymphangiography demonstrating leakage of lipiodol (arrows) from the left wall of the pelvis into the peritoneal cavity.

**Figure 3:**
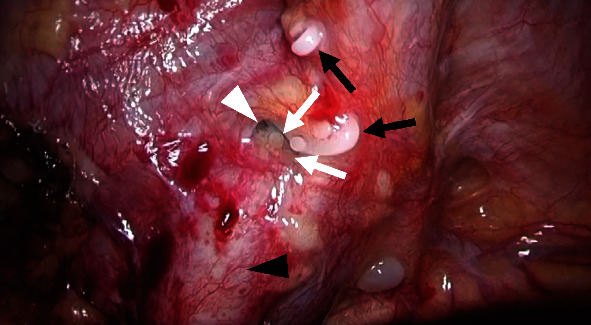
Laparoscopic image taken during surgical repair of lymphorrhea demonstrating droplets of lipiodol (white arrows) flowing out from a dent (white arrowhead) on the left wall of the pelvis; surgical clips (black arrows) that were placed during cystectomy to prevent lymphatic leakage and the left external iliac artery (black arrowhead) can be observed.

**Figure 4:**
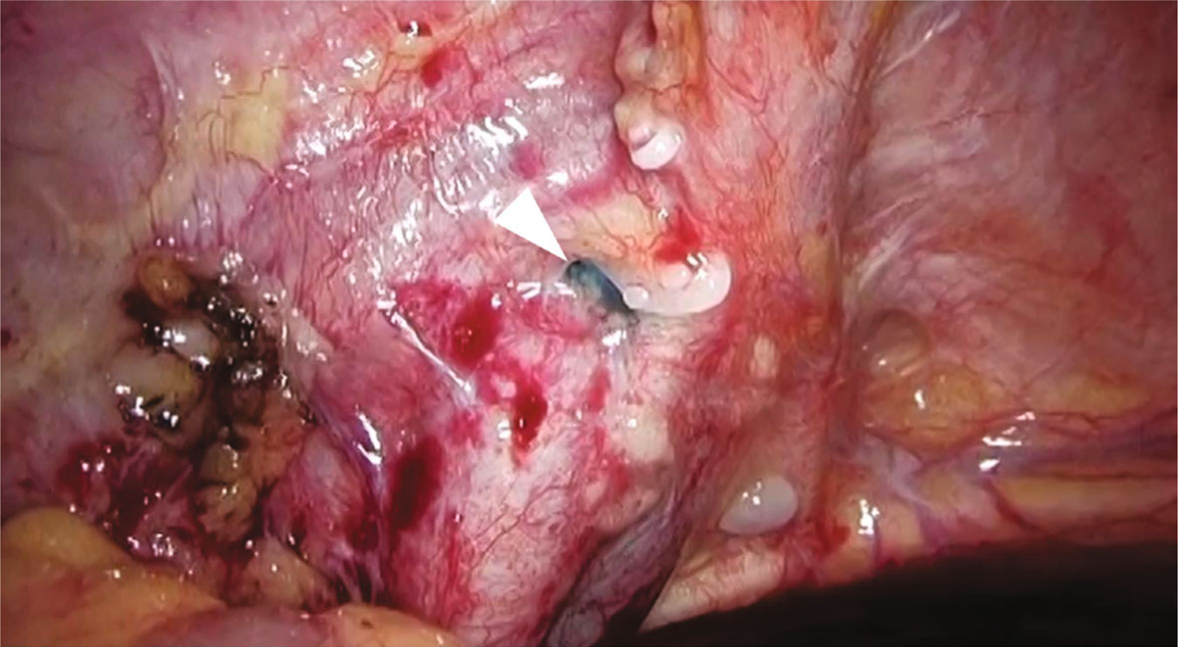
Laparoscopic image taken during surgical repair of lymphorrhea demonstrating indigo carmine (arrowhead) flowing out from a dent on the left wall of the pelvis.

A 79-year-old man presented with high-grade muscle-invasive bladder cancer and underwent robot-assisted laparoscopic radical cystoprostatectomy with urethrectomy, extended pelvic lymph node dissection, and intracorporeal urinary diversion. One week after surgery, clear fluid started leaking from the external urethral orifice. The leakage output was 300 mL per day. CT revealed a medium amount of ascites. Biochemical examination of the ascitic fluid was suggestive of lymphorrhea. A low-fat diet was started, but the leakage output did not decrease. Two months after surgery, intranodal lymphangiography was performed to detect the site of leakage and reduce the amount of lymphatic leakage. Under local anesthesia, bilateral inguinal lymph nodes were punctured with a 23-gauge cathelin needle under ultrasound guidance (Figure 1). Lipiodol was injected slowly under fluoroscopy, showing a network of lymphatic vessels in the pelvis. Lymphatic leakage from the left pelvic wall into the peritoneal cavity was detected (Figure 2). Although the therapeutic effect of lipiodol lymphangiography was expected, the amount of lymphatic leakage did not change after lymphangiography. Thus, laparoscopic surgical repair of lymphorrhea was performed two weeks later. During the repair, a dent was found on the left wall of the pelvis just above the external iliac artery, from which the serous fluid slowly leaked into the peritoneal cavity. Although the dent was suspected to be the lymphatic leakage point, the laparoscopic findings alone were not conclusive. We decided to perform intraoperative lymphangiography to confirm that the dent was the leakage point and to exclude other sites of leakage. A left inguinal lymph node was punctured under ultrasound guidance and lipiodol was slowly injected, showing leakage from the left wall of the pelvis. Then, indigo carmine diluted 10-fold with normal saline was slowly injected. In total, 1 mL of lipiodol and 1 mL of diluted indigo carmine were used. Three minutes after administering lipiodol injection, laparoscopy revealed extravasation of lipiodol and indigo carmine from the dent (Figures 3 and 4 and Supplementary Video). We also confirmed the absence of other leakage sites. The lymphatic leakage point on the left wall of the pelvis was close to the external iliac artery, and suturing the leakage point was a risk factor for vascular injury. Thus, the leakage point was cauterized using an electric scalpel. Additionally, a defect was observed at the pelvic floor region that was sutured at the completion of the previous surgery, and the lymph was considered to have leaked from the external urethral orifice through the defect. The defect was closed with 3-0 Vicryl sutures. No complications were observed. After surgical repair, lymphatic leakage from the external urethral orifice was no longer observed. Abdominal ultrasound revealed a decrease in ascites. One month later, the patient was discharged home.

## 3. Discussion

A small number of studies have reported the usefulness of intraoperative lymphangiography in detecting the leakage point in patients with postoperative lymphorrhea [5–8]. In previous studies, intertoe injection of iso-sulphan blue or indocyanine green, or subcutaneous injection of indocyanine green dye in the inguinal region was performed [5, 7, 8]. In contrast, we performed intranodal lymphangiography in the present case. The clinical outcome of this patient indicates the clinical usefulness of intranodal lymphangiography as it appropriately showed the lymphatic leakage point during surgery.

Intranodal lymphangiography is a relatively new technique, which requires puncture of the inguinal lymph nodes and injection of lipiodol, and it has previously been performed under fluoroscopy guidance to detect lymphatic leakage points [9]. Additionally, this technique is reportedly therapeutically effective for lymphatic leakage [9]. Compared with the conventional pedal lymphangiography, which requires the isolation and cannulation of pedal lymphatic vessels and injection of lipiodol, intranodal lymphangiography is less time-consuming and easier to perform [10].

In patients with postoperative lymphorrhea, the leakage point may be difficult to localize during surgical repair, and unsuccessful surgical repair is not uncommon [11]. Additionally, there can be multiple leakage points, and successful ligation of one leakage point does not always lead to alleviation of the symptoms [6]. The lymphatic system can be clearly visualized on intranodal lymphangiography, and it is also easy to perform during surgical repair. It probably has a higher leakage point detection rate than other methods of lymphangiography.

In conclusion, we report a case in which intranodal lymphangiography made it possible to determine the lymphatic leakage point and to confirm the absence of other leakage points. This technique may be beneficial during surgical repair of lymphorrhea.

## Supplementary Materials

Supplementary Materials Video clip from laparoscopic surgical repair of lymphorrhea demonstrating extravasation of lipiodol droplets from a dent on the left lateral wall of the pelvis.
