# Supplementary material for: Intranodal Lymphangiography during Surgical Repair of Pelvic Lymphorrhea after Radical Cystectomy
Source: Case Rep Urol. 2021 Jul 5;2021:7822422. doi: 10.1155/2021/7822422 (PMC8277509; doi:10.1155/2021/7822422)
Supplement: Supplementary Materials — Video clip from laparoscopic surgical repair of lymphorrhea demonstrating extravasation of lipiodol droplets from a dent on the left lateral wall of the pelvis. [file 7822422.f1.docx]

**Supplementary Materials**

Video clip is available online (https://drive.google.com/file/d/1LupYO-TZhrLzvf3j0E9ZH2SRmOtVkdi4/view?usp=sharing).
